# COVID-19 vaccination acceptance in underserved urban areas of Islamabad and Rawalpindi: results from a cross-sectional survey

**DOI:** 10.1186/s12889-022-14553-3

**Published:** 2022-12-08

**Authors:** Twangar Kazmi, Mujahid Abdullah, Adnan Ahmad Khan, Rana Muhammad Safdar, Sabeen Afzal, Ayesha Khan

**Affiliations:** 1 Akhter Hameed Khan Foundation, Islamabad, Pakistan; 2 Research and Development Solutions, Islamabad, Pakistan; 3 Ministry of National Health Services, Regulations and Coordination (MoNHSRC), Islamabad, Pakistan

**Keywords:** Vaccine inequity, COVID-19, Community immunization, Urban slums, Vaccine willingness

## Abstract

**Background:**

Urban slums are home to a significant number of marginalized individuals and are often excluded from public services. This study explores the determinants of willingness and uptake of COVID-19 vaccines in urban slums in Pakistan.

**Methods:**

The study uses a cross-sectional survey of 1760 respondents from five urban slums in twin cities of Rawalpindi and Islamabad carried out between June 16 and 26, 2021. Pairwise means comparison tests and multivariate logistic regressions were applied to check the associations of socio-demographic factors and COVID-19 related factors with willingness to get vaccinated and vaccination uptake.

**Results:**

Only 6% of the sample was fully vaccinated while 16% were partially vaccinated at the time of survey. Willingness to receive vaccination was associated with higher education (aOR: 1.583, CI: 1.031, 2.431), being employed (aOR: 1.916, CI: 1.423, 2.580), prior infection in the family (but not self) (aOR: 1.646, CI: 1.032, 2.625), family vaccination (aOR: 3.065, CI: 2.326, 4.038), knowing of and living close to a vaccination center (aOR: 2.851, CI: 1.646, 4.939), and being worried about COVID-19 (aOR: 2.117, CI: 1.662, 2.695). Vaccine uptake was influenced by the same factors as willingness, except worriedness about COVID-19. Both willingness and vaccination were the lowest in the two informal settlements that are the furthest from public facilities.

**Conclusions:**

We found low lived experience with COVID-19 infection in urban slums, with moderate willingness to vaccinate and low vaccination uptake. Interventions that seek to vaccinate individuals against COVID-19 must account for urban poor settlement populations and overcome structural barriers such as distance from vaccination services, perhaps by bringing such services to these communities.

**Supplementary Information:**

The online version contains supplementary material available at 10.1186/s12889-022-14553-3.

## Introduction

Vaccination against COVID-19 has emerged as a key preventive intervention. However, the vaccine was identified, developed, tested and rolled out within a year to an unprecedented number of individuals, in effectively the largest vaccination effort in history that is aimed at reaching the entire global population of nearly 8 billion [1]. Many of those that are being approached to be vaccinated, including adults, were unused to being asked to be vaccinated, with such a rapidly developed vaccine [2]. This novelty and rapidity has sometimes led to hesitancy or even mistrust. In addition, speed of the rollout has also meant that that the vaccination process has been unequal and the most marginalized and previously underserved populations have sometimes been left behind [3, 4].

Globally, about 1 billion people live in perennially underserved, densely populated, informal settlements in cities or ‘slums’ [5].Since many of these localities are informal and poorly documented [6], public officials do not know the exact number of individuals they must serve, therefore, public services are under-deployed. In turn, this limits access and availability of public sector services such as clinics or immunization. Mis- or lack of communication, poor quality of services etc. further lower the trust of these citizens of the public sector, further lowering their utilization or demand for such services [7–11]. As a result, some of the most vulnerable populations do not avail many public health services even while living in major cities.

Since 30% of all population and nearly half of all urban population of Pakistan resides in urban slums, the exclusion or inability to reach this large subset of easy-to-reach yet marginalized population became a major concern with the COVID-19 vaccine rollout [12]. The lack of participation of residents from these localities prompted the search to better understand vaccine willingness and hesitancy in such settlements. Although some recent studies have explored the receptivity of COVID-19 vaccines among residents of urban slums in developing countries [13–15], there has not been any local evidence from Pakistan so far.

The present study, which was conducted during June 2021, was one of the earliest studies that examined self-reported experience of residents from five urban and peri-urban poor settlements for prior exposure with COVID-19 and their willingness to receive the vaccine and actual vaccination, considering their socio-demographic and accessibility-related factors. The findings from this study guided the national response.

## Methodology

### Survey design, location and sampling

This is an observational, cross-sectional household survey of 1760 respondents (equally divided between males and females) between June 16 and 26, 2021, in selected areas of Islamabad and Rawalpindi. The selected areas comprised of a mix of dense formal and informal low-income urban settlements with an estimated average household size of 6.2.

Cluster randomization sampling was used for sample size calculation using a UNICEF sample size calculator. Clusters were identified randomly using Google Maps. We assumed a 50% acceptance rate for vaccination, a design effect of 1.5, a relative margin of error at 95% confidence of 0.12 and a 90% response rate, and therefore, the sample size per locality came to 463. This was increased to 480 for all the larger communities (population greater than 30,000), while 160 were recruited from each of the smaller communities (Table 1).

**Table 1:** Location characteristics of study areas

Area	Actual Population	Number of Households	Average Household Size	Sample from each locality	Clusters per locality
Bhara Kahu	125,048	21,123	5.9	480	30
Dhok Hassu	201,212	30,032	6.7	480	30
I-10	44,580	7,984	5.6	480	30
F-7 (France Colony)	9,113	1429	6.4	160	10
G-7 (Low-income Quarters)	29,609	4,707	6.3	160	10
**Total**	**409,562**	**65,275**	**6.2**	**1760**	**140**

We selected these two cities as both had high COVID-19 infection rates and despite being neighboring cities, Rawalpindi was missing out on COVID-19 vaccination due to certain barriers that needed to be understood at that time to increase vaccination uptake. Our sample areas consisted of I-10 (a middle-class locality), G-7 (Low-income but formal locality), F-7 (France Colony, partly formal settlement), Bhara Kahu (low to middle income, completely informal, recent settlement), and Dhok Hassu (low income, long stand informal locality). Populations, number of households and average household size were retrieved from population census 2017 of Pakistan [16].

Only one respondent per household (either male or female) was surveyed. The respondents were all 18 years or older, which at the time was the minimum age to get vaccinated. The survey questionnaire comprised of 38 mostly closed-ended questions. The survey was administered in Urdu (local) language. Informed consent was taken from respondents prior to surveying.

### Data collection and quality assurance

Prior to field implementation, the survey was pilot-tested among 30 respondents (15 females and 15 males) with assumed similar characteristics to our sample, and the questionnaire was improved where issues arose. Data collection was directly administered in-field on electronic tablets by a team of enumerators. The Computer-Assisted Personal Interviews (CAPI) software tool SurveyCTO was used for data collection. Data was monitored in real-time for quality assurance using the SurveyCTO dashboard tool. Quality measures included location, completeness, duration of interviews, and appropriateness of responses. Non-response rate was 2% of the surveyed households.

### Statistical analysis

Stata 16 Software package was used for analysis. First, we calculated descriptive statistics for socio-demographic characteristics and COVID-19 related variables. We conducted two types of analyses to observe the associations. First, we ran pairwise means comparison tests to check for any significant differences between different categories of socio-demographic characteristics for self-reported COVID-19 infections, willingness to get vaccinated, registrations for vaccination, and vaccination uptake.

Then, we applied multivariate logistic regression analyses with forward stepwise selection method using cluster-adjusted standard errors to analyze the associations of willingness to get vaccinated and vaccination uptake with socio-demographic characteristics (sex, age, location, ethnicity, education level, and employment status), past COVID-19 infections to self and family, vaccination uptake of family members, distance from nearest COVID-19 Vaccination Center (CVC), and risk perception of COVID-19. These variables were included in the model as their univariate analyses showed p-values of less than 0.3.

In post-estimation diagnostic tests, both models were tested for correct specification using Stata’s *linktest* command. Pearson chi-squared and Hosmer–Lemeshow chi-squared tests were applied for testing model fits. In order to check for multicollinearity between variables, variance inflation factors (VIF) were calculated.

### Multivariate logistic regression models

Model 1: Willingness to vaccinate

$$Log\ odds=log\left(\frac{P}{1-P}\right)= \alpha +{{\varvec{Z}}}_{{\varvec{i}}}^{\boldsymbol{^{\prime}}}\vartheta + {{\varvec{X}}}_{{\varvec{i}}}^{\boldsymbol{^{\prime}}}\gamma + {\varepsilon }_{i}$$

where *P* = Probability of success = 1 if willing to get vaccinated and 0 otherwise. The index *i* denotes each respondent, ${{\varvec{Z}}}^{\boldsymbol{^{\prime}}}$ is a vector for sociodemographic characteristics, ***X’*** is a vector representing COVID-19-related factors: past own (*X*_*OC*_) and family (*X*_*FC*_) experience with COVID-19, family COVID-19 vaccination uptake (*X*_*FV*_), distance to CVC (*X*_*D*_) and risk perception of COVID-19 (*X*_*RP*_). ${\varepsilon }_{i}$ is the random error term representing the effect of variables omitted from the model. These variables along with their coding are described in Supplementary Material (Table S1).

Model 2: Vaccination uptake

$$Log\ odds=log \left(\frac{P'}{1-P}\right) = \alpha + \boldsymbol{Z}^{\boldsymbol{\prime}}_{\boldsymbol{i}}\vartheta + \boldsymbol{X}^{\prime}_{\boldsymbol{i}}\gamma + \varepsilon_{i}$$

where *P’* = Probability of success = 1 if at least partially vaccinated and 0 otherwise, all other coefficients are explained in regression model 1.

### Variables transformation

The dependent variable of model 1 was willingness to vaccinate for COVID-19 vaccine. It was originally a Likert scale question with possible responses of ‘strongly willing’, ‘willing’, ‘uncertain’, ‘unwilling’ and ‘strongly unwilling’. It was transformed into a dichotomous variable where success was defined as willing (for choices ‘strongly willing’ and ‘willing’) and failure otherwise (for choices ‘uncertain’, ‘unwilling’ and ‘strongly unwilling’).

For model 2, the dependent variable was vaccination uptake of COVID-19 vaccine. This originally had four choices: 1) Only registered, 2) Unvaccinated and unregistered, 3) partially vaccinated, and 4) fully vaccinated. It was converted into a dichotomous variable for logistic regression analysis where success was measured if respondent was at least partially vaccinated (choices 3 and 4) and failure otherwise (choices 1 and 2).

## Results

### Socio-demographic characteristics

The surveyed population is representative of the individual localities that were surveyed, with equal representation of males and females. The mean age was 37.3 with a similar distribution across all localities. Most respondents were ethnically Punjabi or Pushto, while literacy rates were 65–93% across survey localities (Table 2).

**Table 2:** Socio-demographic and COVID-19 related characteristics

	**Bhara Kahu** ***N* = 480**	**Dhok Hassu** ***N* = 480**	**I-10** ***N* = 480**	**F-7 (France Colony)** ***N* = 160**	**G-7 (Low-income Quarters)** ***N* = 160**	**Total** ***N* = 1760**
**Sex**						
Male	239 (49.9%)	240 (50%)	240 (50%)	81 (50.1%)	80 (50%)	880 (50%)
Female	241 (50.1%)	240 (50%)	240 (50%)	79 (49.9%)	80 (50%)	880 (50%)
**Age**						
17–29	139 (29%)	147 (31%)	155 (32%)	54 (34%)	48 (30%)	543 (31%)
30–39	153 (32%)	148 (31%)	117 (24%)	44 (28%)	43 (27%)	505 (29%)
40–49	111 (23%)	109 (23%)	91 (19%)	29 (18%)	32 (20%)	372 (21%)
50–59	47 (10%)	47 (10%)	56 (12%)	22 (14%)	20 (12%)	192 (11%)
60 +	30 (6%)	29 (6%)	60 (12%)	11 (7%)	17 (11%)	147 (8%)
**Ethnicity**						
Punjabi	244 (51%)	216 (45%)	278 (58%)	156 (98%)	113 (71%)	1007 (57%)
Pushto	77 (16%)	203 (42%)	80 (17%)	1 (1%)	17 (11%)	378 (21%)
Others	159 (33%)	60 (12%)	122 (25%)	3 (2%)	30 (19%)	374 (21%)
**Education**						
None	75 (16%)	166 (35%)	42 (9%)	51 (32%)	11 (7%)	345 (20%)
Up to 12 years	303 (63%)	279 (58%)	210 (44%)	97 (61%)	88 (55%)	977 (56%)
University Degree	102 (21%)	34 (7%)	227 (47%)	12 (7%)	60 (38%)	435 (25%)
**Employment Status**						
Self-employed	92 (19%)	140 (29%)	75 (16%)	15 (9%)	7 (4%)	328 (19%)
Employed	131 (27%)	107 (22%)	110 (23%)	68 (42%)	60 (38%)	476 (27%)
Unemployed	257 (48%)	232 (48%)	289 (61%)	77 (48%)	93 (58%)	948 (54%)
**Lived experience of COVID-19**						
Self	21 (4%)	7 (1.5%)	63 (13%)	3 (2%)	20 (13%)	114 (6%)
Family	34 (7%)	11 (2%)	79 (16%)	10 (6%)	27 (17%)	161 (9%)
**Willingness to vaccinate**						
Willing	274 (57%)	280 (60%)	372 (78%)	124 (78%)	119 (74%)	1169 (67%)
**Vaccination uptake**						
Unvaccinated and unregistered	342 (71%)	364 (79%)	259 (54%)	72 (46%)	88 (55%)	1125 (65%)
Only registered	49 (10%)	57 (12%)	71 (15%)	32 (20%)	15 (9%)	224 (13%)
Partially vaccinated	74 (15%)	31 (7%)	81 (17%)	40 (26%)	45 (28%)	271 (16%)
Fully vaccinated	14 (3%)	7 (1.5%)	69 (14%)	12 (8%)	12 (8%)	114 (7%)

Frequency (%) are reported in the table

### Self-reported COVID-19 experience

Self-reported COVID-19 infections were similar for females and males (6% vs 7%, *p* = 0.416) and between those older than 60 years and below 25 years (7% vs 4%, *p* = 0.883) (Table 3). Self-reported infections were higher among individuals who reported that a family member had been infected than those whose reported no such family exposure (39% vs 3%, *p* < 0.001).

**Table 3:** Results for pairwise means comparisons of the sample

Variables	COVID-19 self-infections	COVID-19 vaccine registrations	Partial vaccination	Full vaccination	Completely unvaccinated
**Gender** (Female vs male)	0.01 (0.416)	-0.01 (0.518)	-0.036 (0.037)	-0.01 (0.411)	0.046 (0.021)
**Age** (> 60 vs < 25)	0.003 (0.883)	-0.036 (0.231)	0.063 (0.048)	0.251 (< 0.001)	-0.314 (< 0.001)
**Family member infected** (No vs Yes)	0.359 (< 0.001)	-0.041 (0.138)	-0.136 (< 0.001)	-0.066 (0.001)	0.202 (< 0.001)
**Locality** (Bhara Kahu vs F-7 France Colony)	-0.025 (0.253)	-0.103 (0.001)	-0.102 (0.002)	-0.048 (0.033)	0.15 (< 0.001)
**Education** (University degree vs no education)	-0.124 (< 0.001)	0.099 (< 0.001)	0.132 (< 0.001)	0.064 (< 0.001)	-0.195 (< 0.001)

Mean differences with p-values in parentheses

### Willingness and registration to vaccinate

Willingness to receive the vaccine was high in all areas, ranging from as low as 57% in Bhara Kahu to 78% in I-10 and F-7 (France colony). However, only 13% of respondents had registered to receive COVID-19 vaccination but had not received any doses yet. Registration rates varied by locality (10% to 20%, *p* = 0.001), but were consistent between different age groups (> 60 years: 11% vs. < 25 years: 9%, *p* = 0.231). Those with no education had a lower registration rate compared to those with a university degree (8% vs 18%, *p* < 0.001) (Table 3).

In regression model 1 (Table 4), a higher education or university degree was statistically associated with willingness to get vaccinated (aOR: 1.583, CI: 1.031, 2.431, *p* = 0.036), as was the awareness about the nearest COVID-19 vaccination center (CVC) (aOR: 2.851, CI: 1.646, 4.939, *p* < 0.001) and being employed (aOR: 1.916, CI: 1.423, 2.580, *p* < 0.001). Having past experience of COVID-19 in the family (aOR: 1.646, CI: 1.032, 2.625, *p* = 0.036) and family members being vaccinated (aOR: 3.065, CI: 2.326, 4.038, *p* < 0.001) were statistically significant determinants of willingness. Respondents who were worried about COVID-19 as a risk towards themselves had significantly increased willingness (aOR: 2.117, CI: 1.662, 2.695, *p* < 0.001).

**Table 4:** Logistic regression odds ratios of willingness to vaccinate and vaccination uptake

	**Model 1** **Willingness to Vaccinate**			**Model 2** **Vaccination Uptake**		
**Variables**	**aOR**	**95% CI**	***p*-value**	**aOR**	**95% CI**	***p*-value**
**Sex (Male)**						
Female	0.795	(0.571, 1.106)	0.174	1.094	(0.696, 1.72)	0.696
**Age (17—29)**						
30—39	1.087	(0.835, 1.414)	0.534	2.169*	(1.382, 3.405)	0.001
40—49	1.363	(0.991, 1.874)	0.056	6.247*	(3.967, 9.837)	0.000
50—59	1.559	(0.999, 2.434)	0.051	10.98*	(7.087, 17.02)	0.000
60 +	2.436*	(1.496, 3.965)	0.000	27.81*	(15.69, 49.29)	0.000
**Location (I-10)**						
G-7 (Low-income quarters)	0.493*	(0.332, 0.731)	0.000	0.659	(0.380, 1.144)	0.138
F-7 (France Colony)	0.876	(0.416, 1.846)	0.729	1.003	(0.579, 1.738)	0.991
Bhara Kahu	0.433*	(0.307, 0.611)	0.000	0.555*	(0.337, 0.916)	0.021
Dhok Hassu	0.737	(0.506, 1.073)	0.112	0.441*	(0.279, 0.697)	0.000
**Ethnicity (Others)**						
Punjabi	1.085	(0.820, 1.434)	0.569	0.902	(0.627, 1.296)	0.576
Pushto	1.319	(0.882, 1.971)	0.177	0.989	(0.633, 1.544)	0.960
**Education (None)**						
Up to 12 years	1.092	(0.788, 1.511)	0.598	1.200	(0.781, 1.846)	0.405
University Degree	1.583*	(1.031, 2.431)	0.036	2.000*	(1.146, 3.490)	0.015
**Employment (Unemployed)**						
Self-employed	1.376	(0.987, 1.917)	0.060	1.099	(0.690, 1.750)	0.690
Employed	1.916*	(1.423, 2.580)	0.000	3.403*	(2.299, 5.037)	0.000
**Self-reported infection (No)**						
Yes	1.277	(0.690, 2.366)	0.436	1.203	(0.683, 2.120)	0.522
**Family infection (No)**						
Yes	1.646*	(1.032, 2.625)	0.036	1.789*	(1.008, 3.175)	0.047
**Family vaccination (No)**						
Yes	3.065*	(2.326, 4.038)	0.000	8.294*	(5.238, 13.14)	0.000
Not living with family	0.618*	(0.383, 0.997)	0.048	1.178	(0.332, 4.176)	0.800
**Distance from CVC (don’t know the distance)**						
Less than 1 km	2.851*	(1.646, 4.939)	0.000	6.107*	(2.792, 13.36)	0.000
1–2 kms	1.901*	(1.24, 2.916)	0.003	3.508*	(1.984, 6.204)	0.000
2 + kms	2.280*	(1.721, 3.019)	0.000	3.579*	(2.228, 5.750)	0.000
**Risk perception of COVID-19 (Unworried)**						
Worried	2.117*	(1.662, 2.695)	0.000	1.146	(0.757, 1.732)	0.520
Uncertain	0.818	(0.546, 1.225)	0.330	1.176	(0.652, 2.121)	0.590
**Constant**	0.470*	(0.267, 0.826)	0.009	0.0044*	(0.002, 0.011)	0.000
**Pseudo R2**	0.172			0.365		
**Number of clusters**	110			110		
**Observations**	1,682			1,675		

Base categories are in parentheses. Cluster-adjusted standard errors were used

^*^ Values significant at *p* < 0.05

### Vaccination uptake

**Fig. 1:**
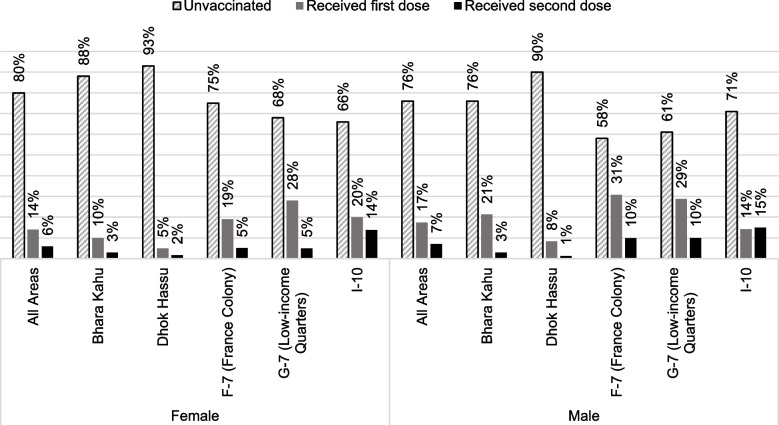
Vaccination uptake by sex and locality

Self-reported full vaccination coverage was 7% across the sample and varied from 1.5% in Dhok Hassu, to 3% in Bhara Kahu, 8% in F-7 (France Colony) and G-7 (Low-income quarters), and 14% in I-10 (Table 2). Partial vaccination was 16% and slightly higher for males (17% vs. 14%, *p* = 0.037) (Table 3). Completely unvaccinated proportion also varied from 58 to 93% in study localities (Fig. 1) and was slightly more among females than males (80% vs. 76%, *p* = 0.021).

Model 2 shows that vaccination increased with age (in part this is consistent with phased opening of vaccination, from older groups down), having a university degree (aOR: 2.000, CI: 1.146, 3.490, *p* = 0.015), being employed (aOR: 3.403, CI: 2.299, 5.037, *p* < 0.001), a history of infection (aOR: 1.789, CI: 1.008, 3.175, *p* = 0.047) or vaccination in the family (aOR: 8.294, CI: 5.238, 13.14, *p* < 0.001) and knowing about a vaccination center (rates doubled if it was within one kilometer of their home). Prior self-reported infection with COVID-19 or being worried about COVID-19 was not associated with vaccination. The odds of vaccination decreased in the two informal settlements of Bhara Kahu and Dhok Hassu (Table 4).

Goodness-of-fit tests of Pearson chi-squared (*p* = 0.239) and Hosmer–Lemeshow chi-squared (*p* = 0.579) indicate that the model 1 fits the data well, and a mean VIF of 1.25 indicates that there is no collinearity between variables. Pearson chi-squared (*p* = 0.731) and Hosmer–Lemeshow chi-squared (*p* = 0.265) tests for model 2 indicate that it fits well, and the mean VIF of 1.28 suggests no collinearity.

## Discussion

We found low self-reported experience of COVID-19 infections (< 13%) in urban slum communities, irrespective of sexes and age groups. There was considerable willingness to receive the vaccine in settlements located near the city-centers and it correlated with increasing age, higher education, employment, an infection in the family, but not for self, family members being vaccinated, being concerned about risks from COVID-19 and knowing where a vaccination center was located. Actual vaccination uptake followed a similar pattern.

Our findings are consistent with international experience from urban slums of Brazil, India and Bangladesh in that higher education and employment are consistent with receptivity of COVID-19 vaccines [15, 17, 18], while effect of age was varied. In the Brazil study younger individuals were more hesitant, while the reverse was true in India. For our purpose, we use definition of hesitancy as a delay or refusal to receive vaccination despite availability of vaccination services [19]. Sex and ethnicity of respondents did not affect willingness or uptake, as was seen in Brazil [15]. In any case, it appears that these social determinants may be context dependent, and specific context such as which age or ethnic group was first included in vaccination, or which group had the most and visible burden of disease etc. may affect perceptions, attitudes and uptake of vaccines differently across locations and societies.

A key element of this context is the “lived experience” with COVID-19, albeit with some nuance. The experience of having encountered COVID-19 in a family member, but not for self, was a significant driver of willingness and vaccine uptake. Some of the hesitancy may be attributed to the low ebb of the epidemic at the beginning of the vaccination drive. In Pakistan, the staged rollout of vaccination by age [20–23] may also have diminished uptake of the vaccination for younger adults in our sample. COVID-19 average positivity rates were 2.2% in Rawalpindi and 1.4% in Islamabad at the time of this survey. Since acceptance of vaccines is correlated with their uptake, vaccination rates were 30% in low-income settlements in our study, while the overall coverage was 47% for Islamabad as a whole at the time. Similarly, 7.9% vaccination rates in Dhok Hassu were considerably lower than 12.9% average for Rawalpindi city at the time.

The low levels of lived experience of COVID-19 infections in our study areas (ranging from 2–13%) are consistent with the observation throughout the response that the epidemic appeared to have been concentrated in the more affluent areas of major cities [24]. While it is possible that less affluent communities may have had less testing, and therefore fewer diagnoses, since around 40% of all tests nationwide have been among symptomatic patients, causality may have run in the opposite direction in that, lower lived experience with the infection also led to seeking of fewer tests in such communities [25]. Our finding of willingness of around 65% is consistent with surveys from Pakistan in other periodsand from the region, such as Bangladesh, where overall vaccine acceptance was 75% nationwide and 58% among slum-dwellers. Other global reports have shown lower levels [17, 26, 27].

A key finding from our study is that both hesitancy and uptake of vaccines were profoundly affected by limited access of these residents to vaccination centers or information about the infection or services, as seen in the two informal settlements, Bhara Kahu and Dhok Hassu, when compared to ones that were closer to vaccination centers. This is consistent with the definition of “low social capital” by Ticona et al. [15].

At the time of the study, the few vaccination centers in these cities were all clustered around city centers, which essentially meant that poor distant communities with limited available time, access to transport, and awareness of public services were effectively excluded from vaccination [11]. On the other hand, simply knowing where a vaccination center was located more than tripled the odds of seeking vaccination, while living within a kilometer of a vaccination increased these odds six-fold. This is consistent with findings from Iran showing high correlation between accessibility and vaccination [28], or from India where presence of health facilities within 2 km doubled the likelihood of child immunization [29]. Even beyond COVID-19 vaccination, a correlation between uptake of health services and distance from facilities is well documented [30–32].

### Strengths and limitations

This study was one of the earliest studies that looked at drivers of COVID-19 vaccine hesitancy and uptake in urban slums of Pakistan, when overall vaccination rates had dropped in the country and new avenues were needed to be explored. However, it has a few limitations. The study is from only two cities and is therefore, not nationally representative. There may be regional differences that can change at least some of the impact of individual social determinants, at least on acceptance. There is also a possibility of recall bias in some questions such as self-reported COVID-19 infections. Finally, almost all of the survey interviews were conducted during day light; hence a proportion of well-off, employed people could have been missed out, resulting in potentially more vaccine hesitancy in the sample.

## Conclusions

Our findings highlight urban slums as a significant location within cities where COVID-19 vaccination rates are low. Older people, employed, highly educated, and those with family members infected or vaccinated are more willing to vaccinate. Locally contextualized concentrated campaigns to raise awareness may help, particularly if supported by local actors. A strong determinant of vaccination is access and thus, bringing vaccination facilities to these localities may help enhance vaccination uptake.

## Supplementary Information

**Additional file 1:** **Table S1. **Names,descriptions and coding of covariates.

## Data Availability

The dataset analysed during the current study is not publicly available because it contains potentially identifying information and was collected based on the condition that respondents’ personal information and details will not be identifiable or shared and will be kept strictly confidential. The dataset is however available from the corresponding author on reasonable request.

## Abbreviations

CVC: COVID-19 Vaccination Center
CAPI: Computer-Assisted Personal Interviews
