# Supplementary material for: COVID-19 vaccination acceptance in underserved urban areas of Islamabad and Rawalpindi: results from a cross-sectional survey
Source: BMC Public Health. 2022 Dec 8;22:2299. doi: 10.1186/s12889-022-14553-3 (PMC9733198; doi:10.1186/s12889-022-14553-3)
Supplement: Supplementary file 1 — Additional file 1: Table S1. Names,descriptions and coding of covariates. [file 12889_2022_14553_MOESM1_ESM.docx]

**Table S1. Names, descriptions and coding of covariates**

| Variable Name | Description | Coding |
| --- | --- | --- |
| Female | Binary variable indicating the gender of the respondent | Male = 1 (base category) Female= 2 |
| Age | Variable depicting age groups in five categories | 18-29 (base category) 30-39 40-49 50-59 60+ |
| Location | Study area of the respondent | I-10 (base category)  G-7 (Low-income quarters)  F-7 (France Colony)  Bhara Kahu  Dhok Hassu |
| Ethnicity | Ethnicity of respondent divided into three heads | Punjabi = 1 Pushto = 2 Others = 3 (base category) |
| Education | Education level of respondent divided into three categories | None = 0 (base category) Up to 12 years = 1 University degree = 2 |
| Employment | Variable representing the employment status | Self Employed = 1 Employed = 2 Unemployed = 3 (base category) |
| Self-Reported infection | Binary variable showing history of own self-reported infection of COVID-19 | Yes = 1 No = 2 (base category) |
| Family infection | Binary variable showing if any member of family ever got infected with COVID-19 | Yes = 1 No = 2 (base category) |
| Family vaccination | Categorical variable telling if any member of the household received at least one dose of COVID-19 vaccination | Yes = 1 No = 2 (base category) Not living with family = 3 |
| Distance form CVC | Categorical variable representing the distance of respondent from a COVID-19 vaccination center | Less than 1 Km = 1  1-2 kms = 2 2+ kms = 3 Don’t know the distance = 4 (base category) |
| Risk Perception of COVID-19 | Variable indicating how worried respondents are about contracting COVID-19 | Worried = 1  Uncertain = 2  Unworried = 3 (base category) |
